# Report of a Case of Fatal Respiratory Obstruction from Softened Mediastinal Glands

**Published:** 1874-07

**Authors:** Charles A. Peabody

**Affiliations:** Worcester, Mass.


ART. II.—*Report of a Case of Fatal Respiratory Obstruction Caused by Softened Mediastinal Glands. A Case of an Extraordinary Discharge of Lumbrici.* By CHARLES A. PEABODY, A. M., M. D., of Worcester, Mass.*

I was called, late in the evening, to see a boy "in a fit." On my arrival I found the boy dead. He was twelve years of age, and I obtained the following history from the mother:

For about a week he had been ailing, and had been kept from school. The cervical glands on both sides were much enlarged, and he seemed to be suffering from a cold, with cough and difficulty of breathing. On Wednesday evening the dyspnœa was very much increased, and there was some spasms, all of which, however, soon passed off. This was repeated on Friday evening with the same result. Saturday was a bad day for him. Sunday he seemed much better than on any day during the previous week and went to bed at nine o'clock. At 10:30, however, he got up in great alarm, saying that his heart had stopped: the dyspnœa was very severe, and gasping or inspiratory: there seemed to be no expiration. He drank some water, but without relief, and strong spasm soon came on. Up to this time no physician had seen him. I was now called and arrived a few minutes before 11 P. M., but too late to see the boy alive.

He had been a healthy, though delicate, child, was easily overcome by exertion, and he would also at times complain of pain about the heart. He was not a cyanosed baby; was very quick to learn. The mother expressed the opinion that the cause of his death was "scrofula humour."

*Autopsy.* Thirty-six hours after death permission was had to examine the chest and throat, (larynx).

There was found binding down the sternum, and intimately adhering to it and the pericardium, a growth almost cartilaginous in consistence, about four inches long by two and one-half wide, and one inch thick. It was supposed to be an hypertrophied thymus gland. The mediastinal glands were all much enlarged and some of them were much softened, while some were quite firm and solid.

---

* The following is extracted from private correspondence and published with the writer's permission.

The trachea was divided and the lungs removed. With some difficulty, as the mouth was tightly closed, the larynx was taken out. It was found to be healthy; but a few specks of curdy material were seen lying loosely on the mucous membrane, which led to examination of the lungs. Here was the cause of death. The bronchial tubes were stuffed with softened, cheesy matter, which, to the eye, the touch and microscope, seemed to be identical with that composing the softened glands. The trachea being somewhat lacerated, no ulcerated opening into it was found, but no one present at the autopsy doubted that such an opening had existed, and that softened glands had discharged into the air passages.

The foramen ovale of the heart was closed only by a valve, and there were traces of an old pericarditis, with some dilatation of the lower part of the right ventricle. Had these enlarged glands, long existing, impeded pulmonary circulation?

In the *London Medical Times* for Jan. 10th, 1874, a case somewhat similar to this is reported. A child aged four, precocious and delicate, suffered from occasional attacks of dyspnœa of doubtful cause. The child died in a fit. *Post mortem* showed the mediastinal and cervical glands enlarged and softened. Close above the left bronchus was an ulcerated opening in the trachea, through which a cheesy substance passed into the air passages causing fatal obstruction. Such cases are not common, at least reports of them are not at all numerous.

A *Case of Worms* that I think of reporting, came to me at the Dispensary.—An Irish girl ten years old, has always lived well, doesn't like potatoes: her abdomen became very protuberant, and hard as a board. She became emaciated. One morning a lumbricoid worm about eight inches in length, jumped out of her mouth. She took "wormifuge," and the result was astonishing.

The mother of this girl showed me a sight that I shall never forget,—a nest of worms that had been passed at one time. I should think there were fifty, or more, of them, and they were from six to twelve inches in length. This quantity she passed twice, and sometimes three times a day, for eight days. On the ninth day she passed, her mother said, "only thirty-four." On the tenth day they were too numerous to count, and were measured. They filled a block tin wash basin, and probably there were not less than *three quarts* of them.

I should think, for I cross-questioned the woman very closely, that the girl passed at least a thousand. This is the third and most severe similar attack the girl has had.

This story may seem gross exaggeration, and especially as I am not absolute in my statements. But the mother of the girl seemed to be intelligent and honest, and to the same questions put in various ways, and on different occasions, returned substantially the same answers. I have no doubt the girl passed at the least 500 lumbrici from six to twelve inches long, within ten days.

Prof. Dewees says that he was shown 96 which had passed in one day. He says, "the sight was truly appalling," but no more were passed. The largest number recorded is in Hunter's case, where 200 were passed in one week.

This case of mine would have peculiar interest, I think, to a certain quack in this city, whose advertisement I enclose, who treats worms and humanity with the same medicine.

**H. C. Champlin, M. D's**

Worm Remedy Depot! Room, No. 15 Exchange Hotel, Worcester, Mass

The Dr. treats all Chr nic Diseases of humanity, makes specialties of Catarrh, Children and Worms, Humanity-Snakes, Toads and Lizards! Has a large cabinet of creatures removed from humanity. Call and see him. Removes all worms alive. Kills neither worms nor patients. Examinations 50 cents. Office hours, 8 A. M., to 8 P. M.

